# Mapping the Quality and Standardization of Methodological Reports in POCUS Research: A Scoping Review Protocol

**DOI:** 10.1111/jep.70342

**Published:** 2025-12-15

**Authors:** Rosana Aparecida Pereira, Larissa Gleyciani Verdeli, Joao Henrique Ribeiro Machado Silva, Laís Facioli Rosa Moreno da Costa, Fernanda Raphael Escobar Gimenes

**Affiliations:** ^1^ Ribeirão Preto College of Nursing. Avenida Bandeirantes University of São Paulo Ribeirão Preto São Paulo Brazil; ^2^ Ribeirão Preto Medical School. Avenida Bandeirantes University of São Paulo Ribeirão Preto São Paulo Brazil

**Keywords:** methodological standardization, POCUS, Point‐of‐Care Ultrasound, research report, scoping review

## Abstract

**Background:**

Point‐of‐Care Ultrasound (POCUS) has become an essential tool for diagnosis and monitoring across diverse clinical settings, enabling rapid and safe decision‐making. Despite its widespread use, considerable heterogeneity persists in how studies report methodological aspects such as protocol descriptions, operator qualifications, and equipment characteristics. This lack of standardization limits comparability between studies, compromises reproducibility, and hinders the integration of POCUS into clinical guidelines and training curricula.

**Objective:**

To map and critically analyze the methodological aspects of studies using POCUS, with a focus on standardization, quality, and reproducibility of reported procedures.

**Methods:**

This scoping review will follow the JBI guidelines and be reported according to the PRISMA‐ScR checklist. Searches will be conducted in PubMed, Web of Science, Embase, CINAHL, Scopus, LILACS, and the Cochrane Database of Systematic Reviews, without restrictions on language or publication period. We will include original studies applying POCUS in humans, regardless of clinical context. Two reviewers will independently screen titles, abstracts, and full texts, with a third reviewer resolving disagreements. Data will be extracted using a standardized form adapted from JBI, capturing information on study design, population characteristics, ultrasound protocols, operator qualifications, and reported outcomes. Results will be synthesized descriptively and organized according to the I‐AIM framework (Indication, Acquisition, Interpretation, and Medical Decision‐Making), supplemented with tables, conceptual diagrams, and visual representations.

**Discussion:**

Given the variability in POCUS literature, this scoping review will identify patterns, inconsistencies, and gaps in methodological reporting. Although international frameworks, such as I‐AIM provide guidance, adherence remains inconsistent, underscoring the need for more uniform standards. Findings from this review are expected to inform reporting practices, enhance methodological quality, and support the safe and effective incorporation of POCUS into clinical practice.

## 1 Introduction

Point‐of‐Care Ultrasound (POCUS) has become a valuable diagnostic tool across diverse clinical settings, largely due to its capacity to deliver immediate information to support rapid, safe clinical decision‐making [1]. It is defined as the use of ultrasound performed directly by the attending healthcare professional, at the time and place of patient care, for diagnostic or monitoring purposes, and integrated into the traditional physical examination [2].

This approach has been widely adopted across different professional groups, including physicians and, more recently, nurses and physiotherapists, particularly in critical settings such as intensive care units, emergency departments, and pre‐hospital care [3].

The substantial growth of scientific literature on POCUS has underscored the need for greater rigor in standardizing reporting practices. Methodological heterogeneity, coupled with the lack of uniform guidelines for describing procedures, operators, and clinical contexts, limits evidence synthesis, reduces study comparability, and compromises reproducibility of findings [4].

In response, several organizations have developed guidelines and use and reporting of POCUS. The American Institute of Ultrasound in Medicine (AIUM), for instance, emphasizes the importance of proper documentation, recommending written records and accessible image storage for review [2]. Nevertheless, adherence to these guidelines remains inconsistent in the scientific literature, highlighting the need for scoping studies to map and systematize current practices.

A preliminary search in PubMed, the Cochrane Database of Systematic Reviews, and JBI Evidence Synthesis found no published or ongoing systematic or scoping reviews specifically examining reporting practices in POCUS research. While some reviews address the clinical efficacy of bedside ultrasound within specific specialties, none critically assess the quality of standardization of methodological reporting in these studies.

The absence of reviews with this specific focus reinforces the relevance of the proposed study. Incomplete or imprecise reporting hampers knowledge development, undermines reproducibility, and obstructs the safe and effective integration of POCUS into evidence‐based clinical practice [5]. Moreover, the increasing incorporation of POCUS into professional training curricula and care protocols emphasizes the urgency for establishing clear guidelines to support knowledge production and dissemination regarding its use.

A scoping review was selected as the methodological approach, as it is best suited to address broad research questions and map the extent, nature, and characteristics of the existing literature in a given field [6]. Scoping reviews allow the identification of available evidence, clarification of concepts, and delineation of knowledge gaps, making them particularly valuable in emerging fields such as POCUS research. Additionally, their methodological flexibility allows the inclusion of diverse study designs and supports a more comprehensive understanding of how reporting practices have evolved.

## 2 Review Questions

1. What elements of methodological design are most frequently reported in POCUS research?
2. How are POCUS protocols described in published studies?
3. What aspects related to POCUS quality, reproducibility, and interpretation are reported?
4. What patterns or deficiencies exist in the reporting of POCUS studies across different clinical specialties?

## 3 Methods

### 3.1 Objectives

#### 3.1.1 Primary Objective

To map and critically analyze methodological reporting practices in studies involving POCUS.

#### 3.1.2 Secondary Objectives

1. To describe how study design elements are reported, including population, intervention, and operator qualifications.
2. To examine the reporting of ultrasound protocols, with attention to equipment, transducers, and image acquisition procedures.
3. To assess which aspects related to POCUS quality, reproducibility, and interpretation are commonly reported.
4. To identify patterns, inconsistencies, and gaps in methodological reporting across different clinical contexts.

### 3.2 Protocol Registration

This protocol has been registered on the Open Science Framework (OSF) and is available at https://doi.org/10.17605/OSF.IO/E3ZN8 [7]. All methodological modifications introduced throughout its development will be described in the final report of this scoping review.

### 3.3 Study Design

This scoping review will be conducted in accordance with the methodological guidelines of the JBI Collaboration [8], ensuring scientific rigor and transparency throughout all stages. Reporting will follow the Preferred Reporting Items for Systematic Reviews and Meta‐Analysis Protocols – Extension for Scoping Reviews (PRISMA‐ScR) checklist [9].

### 3.4 Research Question

The guiding research question was formulated using the PCC framework — Population, Concept, and Context — which helps define the key components of the investigation. The elements are as follows:

**Population (P):** Studies involving humans in which POCUS was applied as a diagnostic, monitoring, or procedural tool;

**Concept (C):** Studies describing the use of POCUS, with emphasis on methodology, execution, or reporting practices (e.g., exam performance, operator characteristics and training, equipment used, outcomes);

**Context (C):** Studies conducted in any clinical setting (e.g., emergency department, intensive care unit, surgery, primary care).

Based on this, the research question for the review is: “How are methodological aspects of POCUS reported in studies involving humans across clinical settings?”

### 3.5 Eligibility Criteria

This review will include studies involving human participants in which POCUS was applied as a diagnostic, monitoring, or procedural tool. Eligible studies must describe the use of POCUS with emphasis on methodological aspects, execution, or reporting practices, such as how the examination was performed, who performed it, operator training, equipment used, and reported outcomes. We will consider peer‐reviewed original research articles, including randomized clinical trials, cohort studies, case‐control studies, Case Report, cross‐sectional studies, and diagnostic accuracy studies. All clinical settings will be eligible (e.g., emergency departments, intensive care units, operating rooms, and primary care), across all geographic regions, with no restrictions on language (provided translation is feasible) or publication period. Although no formal time limits will be applied, practical considerations will prioritize studies published within the last 10–15 years.

Exclusion criteria will comprise studies not directly addressing the use of POCUS, research focused exclusively on conventional ultrasound outside the point‐of‐care context, and publications such as study protocols, letters to the editor, conference abstracts, and unpublished grey literature (e.g., theses and dissertations without public registration).

### 3.6 Search Strategy

The search will be conducted in the following databases: MEDLINE via PubMed; CINAHL via EBSCOhost; Embase via Elsevier; Cochrane Database of Systematic Reviews via Wiley Online Library; Scopus via Elsevier; Web of Science; and LILACS via the Virtual Health Library (BVS), using controlled descriptors from the Health Sciences Descriptors (DeCS) and Medical Subject Headings (MeSH) (see Frame 1 and Supplemental Material). In this study, POCUS is defined as ultrasound performed at the bedside for immediate diagnostic purposes, serving as an extension of the physical examination and integrated into real‐time clinical reasoning [10]. Unlike conventional ultrasound, it is directed at answering specific clinical questions. In the context of gastric assessment, Perlas et al. (2016) proposed the I‐AIM framework, which encompasses the stages of indication (I), acquisition (A), interpretation (I), and medical decision‐making (M). The authors also highlight the importance of structured documentation of findings, even in urgent situations [10].

**FRAME 1:** PCC Framework Applied to the Research Question and Search Terms.

Elements	Description	Search terms employed
**Population (P)**	Studies involving humans in which POCUS was applied as a diagnostic, monitoring, or procedural tool	(Humans OR patients)
**Concept (C)**	Studies describing the use of POCUS, with emphasis on methodology, execution, or reporting practices (e.g., exam performance, operator characteristics and training, equipment used, outcomes)	(“Ultrasonography, Point‐of‐Care” OR “point‐of‐care ultrasound” OR POCUS OR “bedside ultrasound” OR Ultrasound OR Ultrasonography OR “Diagnostic Ultrasounds” OR Sonography) AND (Methodology OR “Diagnostic Techniques and Procedures” OR “operator training” OR “study conduction” OR “reporting practices” OR “study methodology” OR “Methodological Study” OR “Studies, Methodological” OR Procedures OR “Procedure” OR Techniques OR Technique OR “Report, Evaluation” OR “Use‐Effectiveness” OR “Simulation Training” OR Education OR Teaching)
**Context (C)**	Studies conducted in any clinical setting (e.g., emergency department, intensive care unit, surgery, primary care)	(“Emergency Service, Hospital” OR “Intensive Care Units” OR “Primary Health Care” OR Surgery OR “clinical setting” OR emergency OR ICU OR “primary care” OR surgery)

The search strategy will be validated with the support of a librarian specialized in systematic reviews. No restrictions will be applied regarding language or publication date, and translations will be provided when necessary. All identified references will initially be managed in EndNote®, which will be used for automatic duplicate removal. The records will then be transferred to the Rayyan® platform for blinded screening. Study selection will be carried out independently by two reviewers, based on titles and abstracts, with full texts assessed when preliminary information is insufficient. Any disagreement will be solved by a third reviewer.

As this is a scoping review, a critical appraisal of the methodological quality of included studies will not be conducted. Instead, he focus will be on mapping existing knowledge, identifying gaps in the literature, and exploring the breadth of available evidence on the topic.

The information to be extracted will include bibliographic data such as title, authors, year of publication, and country of origin, as well as methodological characteristics, including study design, research setting, sample size and profile, and the profession of the ultrasound operator. In addition, aspects of POCUS application will be collected according to the I‐AIM model [10].

For the domain of **Indication**, data will include the purpose of POCUS use, whether for diagnosis, monitoring, or procedural guidance. For **Acquisition**, details will be extracted on how the examination was performed, including technique, transducer type, patient positioning, and ultrasound device settings. The **Interpretation** domain will capture information on who performed the exam, the operator's training, and the criteria used to interpret findings from both technical and clinical perspectives. The **Medical decision‐making** domain will address how POCUS findings were applied to guide clinical management, including treatment choices, patient monitoring, and procedural interventions. Other relevant elements to be extracted include the clinical setting (e.g., emergency department, intensive care unit, operating room, or primary care), facilitators and barriers to POCUS implementation, and recommendations from clinical guidelines.

Data analysis will be conducted through descriptive synthesis, structured according to the I‐AIM domains of Indication, Acquisition, Interpretation, and Medical Decision‐Making [10]. Quantitative information, such as the frequency with which specific procedures are reported, will be summarized in tables, while qualitative data will be examined through thematic content analysis. Additionally, visual representations—including flowcharts, conceptual diagrams, and summary figures—will be developed to illustrate recurring patterns, variations in practice, and gaps identified in the included studies.

## 4 Discussion

The standardization and quality of methodological reporting in POCUS research remain major challenges for consolidating this technology as an evidence‐based practice. Although a number of studies involving bedside ultrasound has grown substantially, significant heterogeneity persists in how protocols are described, particularly regarding operators, equipment, training, and clinical context [4, 5]. Such inconsistencies compromise reproducibility, hinder comparisons across studies, and ultimately limit the advancement of scientific knowledge.

Several professional bodies, including the American Institute of Ultrasound in Medicine (AIUM) and the American College of Radiology, have published recommendations emphasizing the importance of proper documentation and structured reporting [1, 2]. Likewise, conceptual frameworks such as the I‐AIM model proposed by Perlas et al. (2016) offer valuable methodological guidance to standardize POCUS use and reporting [10]. Despite these initiatives, evidence suggests that adherence to such guidelines is variable, underscoring the need for systematic mapping of current practices to identify strengths, gaps, and opportunities for improvement [4, 5].

In this context, a scoping review provides an appropriate and rigorous methodological approach, as it enables synthesis of the available evidence, clarification of key concepts, and identification of reporting inconsistencies across the literature [6, 9]. Beyond mapping existing knowledge, this review is expected to inform the development of future recommendations aimed at strengthening methodological quality, enhancing comparability between studies, and guiding the safe integration of POCUS into clinical practice. Furthermore, its findings may contribute to professional training curricula and the design of clinical care protocols, ensuring that POCUS continues to expand as a reliable and evidence‐based tool [3, 7].

By advancing standardization and promoting consistent reporting practices, this review seeks to support the methodological robustness of POCUS research and to foster its effective and safe implementation across diverse clinical settings.

## Author Contributions

**Rosana Aparecida Pereira:** conceptualization, methodology, investigation, formal, analysis, data curation, writing – original draft, visualization, software. **Larissa Gleyciani Verdeli:** methodology, data curation, writing – original draft, visualization, software. **Joao Henrique Ribeiro Machado Silva:** methodology, data curation, writing – original draft, visualization, software. **Laís Facioli Rosa Moreno da Costa:** methodology, data curation, writing – original draft, visualization, software. **Fernanda Raphael Escobar Gimenes:** conceptualization, methodology, investigation, formal, analysis, data curation, writing – original draft, visualization, software.

## Funding

The authors received no specific funding for this work.

## Conflicts of Interest

The authors declare no conflicts of interest.

## Supporting information

Supplemental Material.

## Data Availability

The full review protocol, including search strategies and supplementary materials, is registered and openly available on the Open Science Framework (OSF) at DOI: doi.org/10.17605/OSF.IO/E3ZN8. Upon completion of the review, the extracted data, summary tables, and synthesis will also be made publicly accessible through the same repository. No primary data collection was performed in this study.
