# Supplementary material for: Mapping the Quality and Standardization of Methodological Reports in POCUS Research: A Scoping Review Protocol
Source: J Eval Clin Pract. 2025 Dec 15;31(8):e70342. doi: 10.1111/jep.70342 (PMC12706129; doi:10.1111/jep.70342)
Supplement: Supplementary file 1 — Supplemental Material. [file JEP-31-0-s001.docx]

Supplemental Material. Search strategy and results.

| **Database** | **Search strategy** | **Results** |
| --- | --- | --- |
| PubMed (via Medline) | ("Humans"[Mesh] OR patients) AND ("Ultrasonography, Point-of-Care"[Mesh] OR "point-of-care ultrasound" OR POCUS OR "bedside ultrasound") (Ultrasound OR Ultrasonography OR "Diagnostic Ultrasounds" OR "Ultrasound Imaging" OR "Bedside Test" OR "Bedside Testing" OR "Bedside Technology" OR "Bedside Technologies" OR "Point of Care" OR "Point-of-Care" OR "Point of Care Systems" OR "Point-of-Care Systems" OR "Point-of-Care System" OR "Point of Care System" OR "Point of Care Technology" OR "Point-of-Care Technology" OR "Point of Care Testing" OR POCUS OR "Point-of-care ultrasonography" OR "Point of care ultrasonography" OR "Point-Of-Care Ultrasound" OR "Point Of Care Ultrasound" OR Sonography) AND ("Methodology" OR "Diagnostic Techniques and Procedures" OR "operator training" OR "study conduction" OR "reporting practices" OR "study methodology" OR "Methodological Study" OR "Studies, Methodological" OR "Procedures" OR "Procedure" [Mesh] OR "Techniques" [Mesh] OR "Technique" OR "Report, Evaluation" OR "Use-Effectiveness" OR "Simulation Training" OR "Education" OR "Teaching") AND ("Emergency Service, Hospital" OR "Intensive Care Units" OR "Primary Health Care" OR "Surgery" OR "clinical setting" OR emergency OR ICU OR "primary care" OR surgery) | 1475  documents |
| LILACS (via BIREME) | (db:"LILACS") AND (("ultrassonografia à beira do leito" OR "ultrassom point of care" OR POCUS OR "point-of-care ultrasound")) AND (("seres humanos" OR pacientes OR humans OR patients)) AND (("estudo" OR "pesquisa" OR "relato" OR "protocolo" OR "metodologia" OR "descrição" OR "operador" OR "treinamento" OR "study" OR "research" OR "report" OR "protocol" OR "methodology" OR "description" OR "operator" OR "training")) AND (("emergência" OR "UTI" OR "unidade de terapia intensiva" OR "atenção primária" OR "cirurgia" OR "ambiente clínico" OR "emergency" OR ICU OR "intensive care unit" OR "primary care" OR surgery OR "clinical setting")) | 23 documents |
| Embase (via Elsevier) | (Humans OR patients) AND ("Ultrasonography, Point-of-Care" OR "point-of-care ultrasound" OR POCUS OR "bedside ultrasound" Ultrasound OR Ultrasonography OR "Diagnostic Ultrasounds" OR "Ultrasound Imaging" OR "Bedside Test" OR "Bedside Testing" OR "Bedside Technology" OR "Bedside Technologies" OR "Point of Care" OR "Point-of-Care" OR "Point of Care Systems" OR "Point-of-Care Systems" OR "Point-of-Care System" OR "Point of Care System" OR "Point of Care Technology" OR "Point-of-Care Technology" OR "Point of Care Testing" OR POCUS OR "Point-of-care ultrasonography" OR "Point of care ultrasonography" OR "Point-Of-Care Ultrasound" OR "Point Of Care Ultrasound" OR Sonography) AND (Methodology OR "Diagnostic Techniques and Procedures" OR "operator training" OR "study conduction" OR "reporting practices" OR "study methodology" OR "Methodological Study" OR "Studies, Methodological" OR Procedures OR "Procedure" OR Techniques OR Technique OR "Report, Evaluation" OR "Use-Effectiveness" OR "Simulation Training" OR Education OR Teaching) AND ("Emergency Service, Hospital" OR "Intensive Care Units" OR "Primary Health Care" OR Surgery OR "clinical setting" OR emergency OR ICU OR "primary care" OR surgery) | 500 documents |
| Cochrane Central | (Humans OR patients) AND ("Ultrasonography, Point-of-Care" OR "point-of-care ultrasound" OR POCUS OR "bedside ultrasound" Ultrasound OR Ultrasonography OR "Diagnostic Ultrasounds" OR "Ultrasound Imaging" OR "Bedside Test" OR "Bedside Testing" OR "Bedside Technology" OR "Bedside Technologies" OR "Point of Care" OR "Point-of-Care" OR "Point of Care Systems" OR "Point-of-Care Systems" OR "Point-of-Care System" OR "Point of Care System" OR "Point of Care Technology" OR "Point-of-Care Technology" OR "Point of Care Testing" OR POCUS OR "Point-of-care ultrasonography" OR "Point of care ultrasonography" OR "Point-Of-Care Ultrasound" OR "Point Of Care Ultrasound" OR Sonography) AND (Methodology OR "Diagnostic Techniques and Procedures" OR "operator training" OR "study conduction" OR "reporting practices" OR "study methodology" OR "Methodological Study" OR "Studies, Methodological" OR Procedures OR "Procedure" OR Techniques OR Technique OR "Report, Evaluation" OR "Use-Effectiveness" OR "Simulation Training" OR Education OR Teaching) AND ("Emergency Service, Hospital" OR "Intensive Care Units" OR "Primary Health Care" OR Surgery OR "clinical setting" OR emergency OR ICU OR "primary care" OR surgery) | 25 documents |
| CINAHL (via EBSCOhost) | (Humans OR patients) AND ("Ultrasonography, Point-of-Care" OR "point-of-care ultrasound" POCUS "bedside ultrasound" Ultrasound Ultrasonography "Diagnostic Ultrasounds" OR Sonography) AND (Methodology OR "Diagnostic Techniques and Procedures" OR "operator training" OR "study conduction" OR "reporting practices" OR "study methodology" "Methodological Study" OR "Studies, Methodological" OR Procedures OR "Procedure" OR Techniques OR Technique OR "Report, Evaluation" OR "Use-Effectiveness" OR "Simulation Training" OR Education OR Teaching) AND ("Emergency Service, Hospital" OR "Intensive Care Units" OR "Primary Health Care" OR Surgery OR "clinical setting" OR emergency OR ICU OR "primary care" OR surgery) | 258 documents |
| Scopus (via Elsevier) | TITLE-ABS-KEY ( ( Humans OR patients ) AND ( "Ultrasonography, Point-of-Care" OR "point-of-care ultrasound" OR POCUS OR "bedside ultrasound" Ultrasound OR Ultrasonography OR "Diagnostic Ultrasounds" OR "Ultrasound Imaging" OR "Bedside Test" OR "Bedside Testing" OR "Bedside Technology" OR "Bedside Technologies" OR "Point of Care" OR "Point-of-Care" OR "Point of Care Systems" OR "Point-of-Care Systems" OR "Point-of-Care System" OR "Point of Care System" OR "Point of Care Technology" OR "Point-of-Care Technology" OR "Point of Care Testing" OR POCUS OR "Point-of-care ultrasonography" OR "Point of care ultrasonography" OR "Point-Of-Care Ultrasound" OR "Point Of Care Ultrasound" OR Sonography ) AND ( Methodology OR "Diagnostic Techniques and Procedures" OR "operator training" OR "study conduction" OR "reporting practices" OR "study methodology" OR "Methodological Study" OR "Studies, Methodological" OR Procedures OR "Procedure" OR Techniques OR Technique OR "Report, Evaluation" OR "Use-Effectiveness" OR "Simulation Training" OR Education OR Teaching ) AND ( "Emergency Service, Hospital" OR "Intensive Care Units" OR "Primary Health Care" OR Surgery OR "clinical setting" OR emergency OR ICU OR "primary care" OR surgery ) ) | 1589  documents |
| Web of science | (Humans OR patients) AND ("Ultrasonography, Point-of-Care" OR "point-of-care ultrasound" POCUS "bedside ultrasound" Ultrasound Ultrasonography "Diagnostic Ultrasounds" OR Sonography) AND (Methodology OR "Diagnostic Techniques and Procedures" OR "operator training" OR "study conduction" OR "reporting practices" OR "study methodology" "Methodological Study" OR "Studies, Methodological" OR Procedures OR "Procedure" OR Techniques OR Technique OR "Report, Evaluation" OR "Use-Effectiveness" OR "Simulation Training" OR Education OR Teaching) AND ("Emergency Service, Hospital" OR "Intensive Care Units" OR "Primary Health Care" OR Surgery OR "clinical setting" OR emergency OR ICU OR "primary care" OR surgery) | 199  documents |

**Font**: authors
